# Human metapneumovirus: an emerging public health threat and harbinger of a new pandemic

**DOI:** 10.3205/dgkh000607

**Published:** 2025-12-05

**Authors:** İklil Ayberk Saraç, Süleyman Utku Uzun

**Affiliations:** 1 Department of Public Health, Medical Faculty, Pamukkale University, Denizli, Türkiye; 2 Epidemiology Division, Department of Public Health, Medical Faculty, Pamukkale University, Denizli, Türkiye

**Keywords:** human metapneumovirus, emerging pathogen, respiratory infections, epidemiology, public health surveillance, pandemic preparedness

## Abstract

Human metapneumovirus (hMPV), first identified in 2001, has increasingly been recognized as a significant cause of acute respiratory tract infections worldwide. Although often overshadowed by respiratory syncytial virus (RSV) and influenza, hMPV contributes substantially to the global burden of respiratory disease, particularly among young children, older adults, and immunocompromised populations. The COVID-19 pandemic further altered the epidemiology of respiratory viruses, disrupting established seasonal patterns and creating immunity gaps that have fueled unusual hMPV outbreaks in recent years. Despite its clinical relevance, hMPV remains underdiagnosed due to limited awareness, restricted access to reliable diagnostic tools, and frequent co-infections that obscure its contribution to disease severity. Currently, no licensed antivirals or vaccines exist, leaving supportive care as the only treatment option. Ongoing research into monoclonal antibodies, vaccine candidates, and combined RSV–hMPV preventive strategies offers promise but remains in early stages.

In the context of global interconnectedness and the demonstrated capacity of respiratory viruses to cause widespread disruption, hMPV raises important concerns as a potential emerging public health threat. While unlikely to cause pandemic-scale disruption, hMPV’s endemic circulation and disproportionate impact on vulnerable populations warrant its recognition as an emerging threat demanding proactive public health intervention and sustained investment in prevention strategies. This review examines hMPV’s evolving role as a public health threat in the post-COVID-19 landscape, where altered epidemiological patterns and increased surveillance have highlighted its underappreciated impact.

## Abstract (de)

Das humane Metapneumovirus (hMPV), das erstmals 2001 identifiziert wurde, wird weltweit zunehmend als bedeutende Ursache für akute Atemweginfektionen anerkannt. Obwohl es oft vom Respiratorischen Synzytial-Virus (RSV) und Influenzaviren überschattet wird, trägt das hMPV erheblich zur globalen Belastung durch Atemwegerkrankungen bei, insbesondere bei Kleinkindern, älteren Erwachsenen und immungeschwächten Bevölkerungsgruppen. Die COVID-19-Pandemie hat die Epidemiologie von Atemwegsviren verändert, etablierte saisonale Muster gestört und Immunitätslücken geschaffen, die in den letzten Jahren zu ungewöhnlichen hMPV-Ausbrüchen geführt haben. Trotz seiner klinischen Relevanz wird das hMPV aufgrund des begrenzten Bewusstseins, des eingeschränkten Zugangs zu zuverlässigen Diagnosewerkzeugen und häufiger Koinfektionen, die seinen Beitrag zur Schwere der Erkrankung verschleiern, nach wie vor zu selten diagnostiziert. Derzeit gibt es keine zugelassenen antiviralen Medikamente oder Impfstoffe, sodass die unterstützende Behandlung die einzige Behandlungsoption bleibt. Laufende Forschungen zu monoklonalen Antikörpern, Impfstoffkandidaten und kombinierten RSV-hMPV-Präventionsstrategien sind vielversprechend, befinden sich jedoch noch in einem frühen Stadium.

Vor dem Hintergrund der globalen Vernetzung und der nachgewiesenen Fähigkeit von Atemwegviren, weitreichende Störungen zu verursachen, gibt hMPV Anlass zu großer Sorge als potenzielle neue Gefahr für die öffentliche Gesundheit. Auch wenn es unwahrscheinlich ist, dass hMPV Störungen im pandemischen Ausmaß verursacht, rechtfertigen seine endemische Verbreitung und seine unverhältnismäßigen Auswirkungen auf gefährdete Bevölkerungsgruppen seine Anerkennung als neue Gefahr, die proaktive Maßnahmen im Bereich der öffentlichen Gesundheit erfordert.

## Introduction

The global burden of acute respiratory tract infections (ARTIs) remains a cornerstone challenge for public health systems worldwide. These infections affect individuals of all ages, posing a significant global health burden. In 2021, an estimated 344 million incident episodes of lower respiratory tract infections (LRI) occurred globally. The same year saw 2.18 million deaths attributed to LRIs [1]. A substantial portion of these fatalities, specifically 502,000 deaths, occurred in children younger than 5 years, with 254,000 deaths concentrated in countries with a low socio-demographic index [1]. Despite years of public health efforts and medical advancements, the burden of LRIs remains high, particularly in low- and middle-income countries. This underscores the continuous need for vigilance and research into all respiratory viruses, including less-known pathogens. The spectrum of respiratory pathogens includes numerous viral agents, each with distinct epidemiological patterns and clinical presentations. Understanding this diversity becomes crucial for developing targeted public health interventions and allocating resources effectively. The broad category of “other viral etiologies” suggests that viruses not specifically tested for could contribute significantly to the total burden, potentially leading to an underestimation of the true impact of under-recognized pathogens like hMPV.

The COVID-19 pandemic underscored how novel respiratory pathogens can rapidly provoke global crises. The emergence of SARS-CoV-2 dramatically amplified awareness of respiratory virus threats, exposing critical vulnerabilities in global surveillance, healthcare capacity, and pandemic preparedness. While significant resources have been directed towards understanding and mitigating SARS-CoV-2 and influenza, numerous other respiratory pathogens continue to circulate with substantial, yet often underappreciated, impact. Among these, human metapneumovirus (hMPV) has emerged as a significant pathogen since its relatively late discovery in 2001. Despite causing a substantial proportion of ARTIs across all age groups, hMPV remains shrouded in relative obscurity compared to its counterparts like respiratory syncytial virus (RSV) or influenza. 

This review critically examines hMPV through the lens of contemporary public health, assessing its virological characteristics, global epidemiology, clinical significance, diagnostic and therapeutic limitations, and crucially, evaluating its potential as an emerging threat that warrants heightened vigilance and proactive countermeasures in the post-COVID-19 landscape.

## Increased awareness of respiratory viruses post-COVID-19

The COVID-19 pandemic profoundly altered the epidemiology and seasonality of respiratory viruses [2]. Non-pharmaceutical interventions (NPIs) implemented during the pandemic, such as mask-wearing, social distancing, and lockdowns, not only reduced the spread of SARS-CoV-2 but also led to significant decreases in the circulation of seasonal influenza, respiratory syncytial virus (RSV), and hMPV [3]. These reductions in community infection burden resulted in fewer hospitalizations and deaths associated with non-SARS-CoV-2 respiratory infections [4].

However, with the lifting of NPIs, the activity of most viruses has returned to or even exceeded historical levels, with unusual off-season surges reported, particularly for RSV [4]. hMPV incidence also declined during COVID-19 restrictions but subsequently increased after these measures were removed [5]. This confirms the impact of NPIs on viral spread. In the post-pandemic period, epidemiological shifts for hMPV have been observed, including unusual seasonal patterns, increased co-infection rates, and altered age distributions [6]. Furthermore, the pandemic highlighted the interconnectedness of respiratory virus threats and the necessity for integrated, year-round surveillance systems capable of detecting and responding to all significant respiratory pathogens, not just novel emergents. This context provides a unique opportunity to re-evaluate established but under-prioritized pathogens like hMPV.

## Discovery and history of human metapneumovirus (hMPV)

Human metapneumovirus (hMPV) was first identified in 2001 in the Netherlands by van den Hoogen et al. [7], who isolated it from nasopharyngeal aspirate samples collected from young children. The discovery stemmed from efforts to identify the causative agent in children with unexplained respiratory tract infections where common pathogens like RSV, influenza-, parainfluenza-, and adenovirus had been ruled out. Using molecular techniques (primarily PCR with degenerate primers targeting conserved regions of paramyxoviruses), they identified a novel virus in nasopharyngeal aspirates collected over a 20-year period (1958–2000) from children in the Netherlands. Phylogenetic analysis revealed it belonged to the pneumoviridae family, genus *Metapneumovirus*, distinct from but closely related to avian metapneumovirus (aMPV) and RSV (which belongs to the genus orthopneumovirus within the same family) [7], [8]. 

Retrospective serological studies have since shown that hMPV antibodies were present in humans for at least 50 years prior to its official discovery, with samples from the Netherlands dating back to 1958 [9]. This evidence confirms that the virus had been circulating in human populations long before its formal identification. Based on its morphological, biochemical, and genetic characteristics, hMPV was tentatively classified as a new member of the metapneumovirus genus, due to its similarity with avian metapneumovirus (APV), which was previously the sole member of this genus [7].

hMPV shares significant similarities with respiratory syncytial virus (RSV), its closest human pathogen relative within the pneumoviridae family. Both viruses cause similar clinical syndromes, exhibit comparable seasonal patterns in temperate climates, and primarily affect young children and elderly populations [10]. The clinical similarity of hMPV symptoms to those of other common respiratory viruses like RSV and influenza poses a significant challenge in clinical practice. This clinical overlap often leads to hMPV infections being misdiagnosed or broadly categorized as “viral respiratory illness” if specific laboratory testing is not performed. This obscures the true incidence and burden of the virus, potentially causing diagnostic delays and even unnecessary antibiotic use. Compared to influenza viruses, hMPV demonstrates lower mutation rates and less antigenic drift, suggesting more stable vaccine targets but also indicating persistent circulation without significant evolutionary pressure. Unlike SARS-CoV-2, which emerged as a novel pathogen causing a pandemic, hMPV represents an endemic virus with established population immunity, though this immunity appears incomplete and waning, allowing for reinfections throughout life [11]. 

The fact that hMPV remained undiscovered until 2001 implies that a significant public health burden went specifically unattributed for many years. This “invisible pathogen” status highlights how diagnostic limitations can restrict our understanding of respiratory disease etiology. Challenges such as the virus’s slow growth in cell culture also contributed to the delay in its discovery [12]. This underscores the critical importance of advancements in diagnostic capabilities for identifying previously unknown or overlooked pathogens and understanding their true impact on public health.

## Public health importance of hMPV

Despite hMPV being a significant contributor to global respiratory infections, particularly in the pediatric population, it remains an under-recognized pathogen [13]. This is largely due to limited access to molecular diagnostics and routine surveillance, especially in low- and middle-income countries (LMICs), leading to underdiagnosis [13]. Comprehensive data on the burden of hMPV in adults are still limited, and the scarcity of evidence in community settings, coupled with a lack of routine testing, makes it difficult to estimate the virus’s true burden [14].

Low public awareness of hMPV compared to RSV and influenza can lead individuals to delay seeking healthcare or neglect preventive measures. The scientific literature on hMPV, while growing, remains significantly less extensive than that for RSV or influenza. Many clinicians, particularly outside pediatric and infectious disease specialties, may not routinely consider hMPV in differential diagnoses, and public health messaging rarely highlights it. Furthermore, the absence of an approved antiviral treatment or vaccine for hMPV exacerbates its public health challenge. This creates a vicious cycle where a pathogen remains under-recognized and under-researched. hMPV has been the “Cinderella” of the respiratory virus world, often overlooked despite its significant contribution to respiratory morbidity. When a pathogen is not adequately recognized or routinely tested for, limited data are collected, which in turn perpetuates the perception that the virus is less significant, leading to less funding for research and diagnostics, thus continuing the cycle of under-recognition. This has serious implications for resource allocation and policy development.

The limited access to molecular diagnostics, particularly in LMICs, creates a significant disparity in global health security. Regions that bear the highest burden of respiratory infections often have the lowest capacity to diagnose and monitor hMPV. This leads to a skewed understanding of the virus’s global impact and hinders effective, localized public health interventions. Addressing this inequity requires international collaboration and investment in diagnostic infrastructure.

## Reasons for potential public health threat

hMPV poses several characteristics that warrant its classification as an emerging or persistent public health threat due to its prevalence, morbidity, seasonal variability and co-circulation, diagnostic challenges, potential for severe outcomes and lack of countermeasures.

hMPV is a significant respiratory pathogen that contributes to acute respiratory infections, particularly in vulnerable populations such as children, the elderly, and immunocompromised individuals [1]. It causes a wide range of respiratory illnesses, including bronchiolitis, pneumonia, and asthma exacerbations, leading to substantial hospitalization rates and healthcare costs [15]. Differentiation from other respiratory viruses based on symptoms alone is impossible, and routine testing is not universally implemented, leading to underestimation of its true burden [16]. The morbidity and mortality rates of hMPV are comparable to infection by influenza viruses and RSV [15]. Currently, there are no specific antiviral drugs or vaccines available, meaning disease management largely relies on supportive care [1]. The genetic variability and continuous evolution of the virus, particularly in its G protein, enable it to evade immunity and lead to the emergence of new variants [15].

The resurgence of hMPV in the post-COVID-19 era, coupled with altered seasonal patterns and increased co-infection rates, highlights the dynamic nature of the virus [2]. hMPV exhibits variable seasonal patterns, sometimes peaking in late winter/spring (after RSV/influenza), and frequently co-circulates with other respiratory viruses, complicating diagnosis and burden estimation [17]. Its seasonality can be unpredictable, potentially straining healthcare resources outside typical “flu season”. 

The combination of these factors makes hMPV a significant and potentially growing public health threat. The disproportionate impact on vulnerable populations, the severe outcomes it can cause, the lack of specific treatments and vaccines, and its genetic diversity create a “perfect storm” where a significant pathogen remains inadequately controlled. This justifies why hMPV, despite its long circulation, is now considered a “new public health threat”. The epidemiological uncertainty in the post-COVID-19 era further elevates hMPV’s threat level. The pandemic disrupted the virus’s seasonal patterns, making previously predictable outbreaks more unpredictable. This means that traditional seasonal preparedness strategies (e.g., vaccine campaigns, hospital bed capacity planning) may no longer be sufficient. This unpredictability necessitates that public health strategies rely on more agile and real-time surveillance systems.

## Epidemiology of hMPV

### Global distribution of hMPV cases – most affected regions and age groups

hMPV is a globally distributed pathogen, with evidence of circulation on every continent where surveillance has been conducted [18]. Studies have found hMPV in 5–25% of acute respiratory infections in children, depending on region and season [18], [19]. For example, population-based surveillance in the U.S. detected hMPV in ~4% of hospitalized pediatric ARI cases, with an annual hospitalization incidence around 1–5 per 1,000 children under five [19]. In Australia and Asia, similar rates have been reported (e.g. ~7% in one Australian study) [20]. Serological evidence suggests that nearly every child contracts hMPV by age five [21]. While children dominate the case counts, a nontrivial fraction of severe hMPV infections occur in adults: U.S. data suggest annual hospitalization rates of ~2.2 per 1,000 in adults ≥65, comparable to RSV and higher than influenza [20]. Thus, hMPV adds substantially to the global burden of viral lower respiratory infections in both pediatrics and geriatrics.

### Seasonal patterns

hMPV typically exhibits a seasonal pattern in temperate regions, typically circulates in late winter and spring. Northern Hemisphere surveillance shows hMPV peaks in January–March, often lagging slightly behind the main influenza season [15], [22]. Steinberg et al. [23] noted most cases occurring in winter-spring, with very few in summer. In the Southern Hemisphere, hMPV tends to peak in their winter months. Tropical regions may have less pronounced seasonality, but often see increased cases during rainy seasons. In 2020–2022, COVID-19 mitigation caused an unusual pattern: hMPV virtually disappeared for a season, then re-emerged with delayed or off-season outbreaks once measures were relaxed [15], [23]. This demonstrates that hMPV dynamics are sensitive to social behavior and public health interventions.

## Virus characteristics

hMPV is an enveloped, negative-sense, single-stranded RNA [15]. Two major genetic lineages exist (A and B), each further divided into sub-lineages (A1, A2, B1, B2), which can co-circulate and may exhibit slight differences in antigenicity and potentially disease severity [15].

hMPV primarily spreads through respiratory droplets, direct contact, and surface contamination [7]. According to laboratory research, hMPV can survive for up to 24 to 48 h at room temperature with moderate humidity on nonporous surfaces such as plastic and stainless steel. On the other hand, the virus only lasts a much shorter time on permeable substances consisting of paper or cloth [24]. Although there is limited data on eliminating hMPV from surfaces, the use of alcohol based hand rubs containing at least 60% alcohol is recommended [25].

## Clinical presentation and diagnosis

hMPV infection presents with a wide spectrum of respiratory symptoms, largely indistinguishable from other common respiratory viruses based on clinical features alone. The clinical symptoms of hMPV infection are similar to those caused by RSV infection, ranging from upper respiratory tract disease to severe bronchiolitis and pneumonia [26]. hMPV symptoms in adults often mimic influenza, including persistent cough, fatigue, and mild fever [21]. While hMPV infections are generally mild and self-limiting, they can be complicated in the elderly and immunocompromised patients [21].

Diagnosis of hMPV primarily relies on nucleic acid amplification tests, such as reverse transcriptase polymerase chain reaction (RT-PCR) on respiratory specimens (nasopharyngeal swabs or aspirates) [18]. Multiplex RT-PCR is the most frequently used method, followed by real-time RT-PCR [27]. Viral culture of hMPV is technically possible but not practical clinically, as hMPV grows very slowly and requires specialized cells with trypsin. Some rapid antigen detection tests (RADTs) exist for hMPV, but they are generally less sensitive than PCR, especially in adults where viral loads may be lower [28].

## Risk factors of infection

The incubation period typically ranges from 3 to 5 days [29]. Certain groups are at higher risk of infection and severe illness. Infants and young children are the primary targets: the first infection almost always occurs by age 5 [19]. In particular, children under 2 years old have the highest rates of hMPV-associated hospitalization [18], [19]. The elderly are also disproportionately affected: adults >65 years often suffer pneumonia or exacerbations when infected. Immunocompromised persons (transplant recipients, HIV-infected, chemotherapy patients) have increased risk of disseminated or severe pulmonary hMPV disease [15]. Crowded environments and healthcare facilities are identified as significant environmental amplifiers for viral spread [7].

## Treatment and management

There is currently no specific antiviral therapy for hMPV. The management of hMPV primarily relies on supportive care, with oxygen therapy and hydration being critical interventions [10]. For mild cases, treatment is outpatient: hydration, antipyretics, and nasal suctioning for infants. In more severe pediatric cases (e.g. bronchiolitis), supplemental oxygen and close monitoring are key. There is no licensed antiviral like ribavirin or neuraminidase inhibitors, approved for hMPV. Although ribavirin has in vitro activity, clinical use is not supported by evidence. Therefore, critical care for severe hMPV focuses on respiratory support (ventilation, possibly extracorporeal membrane oxygenation in fulminant cases) and treatment of complications (antibiotics for secondary bacterial pneumonia if indicated).

The lack of targeted therapy poses limitations. Pediatricians often apply general bronchiolitis guidelines (as for RSV) to hMPV cases: bronchodilators or corticosteroids are not routinely recommended, but sometimes tried empirically. For immunosuppressed patients with severe hMPV, case series have explored inhaled or intravenous ribavirin and intravenous immunoglobulin, but benefits are unclear. In the absence of proven drugs, preventing hospital-acquired spread is crucial. Infection control (patient isolation, cohorting, PPE, hand antisepsis, surface disinfection) follows standard respiratory virus protocols.

## Potential impact of hMPV health systems

hMPV leads to significant hospitalization rates and healthcare costs [30]. In 2018, hMPV was estimated to be associated with over 640,000 hospitalizations among children under five globally. In 2019, it was estimated to cause approximately 470,000 hospitalizations globally in adults over 64 years [31]. High rates of ICU admission and mortality are observed in elderly and high-risk adults [13]. Co-infections increase the risk of ICU admission [30]. The economic burden ranges from $3,850 to $9,946 USD per hospitalization in high-income countries [32].

The significant hospitalization rates and associated healthcare costs of hMPV, particularly in vulnerable populations, represent a substantial economic burden that is often “under-recognized”. This hidden economic cost, stemming from factors like prolonged hospital stays, ICU admissions, and supportive care, justifies greater investment in diagnostics, prevention, and specific treatments, as these investments could lead to significant cost savings for healthcare systems in the long run. Also misdiagnosis of viral hMPV infection as bacterial pneumonia leads to unnecessary antibiotic prescriptions, fueling antimicrobial resistance (AMR) – a critical global health threat.

## Significant hMPV outbreaks in recent years

Recent reports of increased hMPV cases from China, India, and Europe highlight the virus’s underestimated role during winter seasons [30]. In early 2025, a 17% increase in hMPV-related pediatric hospitalizations was observed in the US and China compared to 2023, with similar patterns in elderly and immunosuppressed patients [30]. In late 2024, hMPV accounted for 6.2% of respiratory illness tests and 5.4% of hospital admissions in China, surpassing rhinovirus and adenovirus [30]. Scattered cases in India prompted public health advisories, with approximately 90 reported cases as of March 2025 [30].

For instance, after the 2020 lockdowns lifted, Holland and the UK reported summertime spikes in hMPV among children – a time when hMPV normally would be minimal [13]. In Spain, an unusual outbreak occurred in November-December 2021, coinciding with the sixth wave of COVID-19, affecting older patients more severely with increased hypoxia, pneumonia, longer hospital stays, and greater ICU needs [33]. A community hospital in eastern England experienced an outbreak in July-September 2010, with a 29.4% attack rate among confirmed/probable cases, primarily affecting elderly individuals with comorbidities [34].

In late 2023 and early 2024, Chinese health agencies and WHO issued alerts about large clusters of hMPV pneumonia in schools and communities [35]. These clusters included hospitalized children and even adult cases, underscoring that relaxed COVID-era immunity gaps could allow hMPV to spread out of season.

Documented hMPV outbreaks in congregate living settings like long-term care facilities and hospitals demonstrate that these environments are particularly vulnerable to hMPV outbreaks. This is likely due to close contact among susceptible individuals, shared airspaces, and a high proportion of immunocompromised individuals. This implies that infection control protocols in such settings should specifically account for hMPV, similar to how they manage influenza or RSV. The “off-season” outbreaks in the Netherlands and the unusual timing in Spain in the post-COVID-19 era underscore the unpredictable nature of hMPV circulation within the changing epidemiological landscape. This means public health authorities cannot solely rely on historical seasonal patterns for preparedness, requiring more dynamic and real-time surveillance to detect and respond to outbreaks occurring outside typical seasons.

## Pandemic preparedness

While hMPV itself isn’t a pandemic threat, hMPV should be part of pandemic preparedness in a broad sense. Lessons from COVID-19 and other outbreaks underscore the need to strengthen preparedness for respiratory pathogens like hMPV. Integrating hMPV detection into existing national and global respiratory virus surveillance networks (e.g., Global Influenza Surveillance and Response System (GISRS) for influenza, broader platforms) is crucial. Early warning systems (syndromic surveillance, multiplex testing in sentinel sites) can detect unusual spikes. For example, some European networks now include hMPV in their routine panels; this allowed them to notice the 2021/22 atypical season. On a global scale, data-sharing of unusual hMPV activity (like through WHO’s event-based alerts) helps map virus trends.

Preparedness also means educating healthcare workers. During the COVID-19 crisis, many pediatricians became adept at distinguishing viral vs bacterial pneumonia. Similar training for hMPV could improve clinical suspicion. For instance, knowing that hMPV can co-infect with RSV might encourage clinicians to test for hMPV when a child tests negative for RSV/flu. Public health messaging (to clinicians and to the public) should highlight that not all winter pneumonia is influenza or COVID: hMPV and other viruses may be involved. In infection control terms, hospitals should include hMPV in their respiratory isolation policies during respiratory seasons, given its similar transmission to RSV.

## Prevention strategies

Long-term prevention hinges on vaccines and general public health measures. No licensed hMPV vaccine exists yet, but several candidates are in development. Combination vaccines against RSV and hMPV have entered clinical trials [36]. It is noteworthy that the hMPV vaccine development process has not progressed as far as COVID vaccine production [37]. The virus’s ability to evade immune responses and the lack of understanding of long-term immunity remain significant barriers to hMPV vaccine development [37]. Herd immunity is critical in preventing the spread of the virus. Broad vaccination coverage is critical to controlling hMPV in terms of herd immunity, especially among young children, the elderly, and immunocompromised groups [37].

Non-pharmaceutical interventions (masks, hand hygiene) remain important. The COVID-19 experience showed that masks and distancing almost eliminated hMPV spread for a season [7]. Encouraging mask use by sick caregivers and in high-risk settings (e.g., pediatric wards) can reduce hMPV transmission. Every hMPV patient should be placed under droplet precautions to limit and prevent spread. Routine hand antisepsis and surface disinfection interrupt its spread as well [15]. Wiping surfaces at home and proper handwashing are basic precautions for patients and their families. Especially in the absence of vaccines or specific treatments, these basic public health practices form the first line of defense in controlling the spread of the virus and protecting vulnerable populations.

## Public health policies

Comprehensive policies at the national and international levels are required to reduce the impact of hMPV on public health. These policies should include strengthening integrated surveillance systems, increasing access to molecular diagnostics, and accelerating efforts to develop vaccines and antiviral treatments. Inclusion of hMPV in national and global acute respiratory infection action plans would be prudent. Many countries’ respiratory disease policies focus on influenza, RSV, and now COVID-19; adding hMPV would involve ensuring diagnostic capacity and readiness. Internationally, sharing hMPV sequence and outbreak data (as done for influenza) could enable monitoring of new variants. Policies should also address equity: vaccine and drug trials should consider vulnerable groups where hMPV hits hardest. Ultimately, pandemic preparedness frameworks (like WHO’s Pandemic Influenza Preparedness Framework) could name hMPV as a virus of interest, given its broad impact and the fact it has pandemic potential if it were to mutate significantly. Addressing inequalities, particularly in low- and middle-income countries, is critical for global health security. 

## Conclusion

hMPV, although officially identified in 2001, has been a pathogen circulating in human populations for decades and contributing significantly to the global respiratory disease burden. It causes significant morbidity and mortality annually, particularly among infants, the elderly, and immunocompromised individuals, straining healthcare systems worldwide. While hMPV is unlikely to cause an explosive pandemic like COVID-19 due to its low basic reproduction number (R0), its widespread circulation, severe impact on vulnerable groups, and lack of specific countermeasures make it a persistent and silent public health threat.

This review highlights the growing public health significance of hMPV and the urgent need for intervention. Future strategies to reduce the global burden of hMPV and strengthen preparedness for respiratory virus outbreaks should include establishing integrated surveillance systems, disseminating improved and accessible diagnostic tools, and accelerating efforts to develop vaccines and antiviral treatments. Public health professionals should lead these efforts by raising awareness of this virus, educating healthcare workers, and promoting national and international collaboration. Vigilance, research, and investment are key to ensuring hMPV does not evolve from a persistent epidemic threat into a more disruptive force.

## Notes

### Authors’ ORCIDs

- Saraç IA: https://orcid.org/0000-0002-5484-9136
- Uzun SU: https://orcid.org/0000-0002-8876-2848

### Funding

None. 

### Competing interests

The authors declare that they have no competing interests.
